# Evaluation of Oral Antiretroviral Drugs in Mice With Metabolic and Neurologic Complications

**DOI:** 10.3389/fphar.2018.01004

**Published:** 2018-09-04

**Authors:** Fuu-Jen Tsai, Mao-Wang Ho, Chih-Ho Lai, Chen-Hsing Chou, Ju-Pi Li, Chi-Fung Cheng, Yang-Chang Wu, Xiang Liu, Hsinyi Tsang, Ting-Hsu Lin, Chiu-Chu Liao, Shao-Mei Huang, Jung-Chun Lin, Chih-Chien Lin, Ching-Liang Hsieh, Wen-Miin Liang, Ying-Ju Lin

**Affiliations:** ^1^School of Chinese Medicine, China Medical University, Taichung, Taiwan; ^2^Genetic Center, Department of Medical Research, China Medical University Hospital, Taichung, Taiwan; ^3^Department of Biotechnology, Asia University, Taichung, Taiwan; ^4^Section of Infectious Diseases, Department of Internal Medicine, China Medical University Hospital, Taichung, Taiwan; ^5^Department of Microbiology and Immunology, Chang Gung University, Taoyuan, Taiwan; ^6^Molecular Infectious Disease Research Center, Chang Gung Memorial Hospital, Linkou, Taiwan; ^7^Graduate Institute of Biostatistics, School of Public Health, China Medical University, Taichung, Taiwan; ^8^Rheumatism Research Center, China Medical University Hospital, Taichung, Taiwan; ^9^Graduate Institute of Natural Products and Research Center for Natural Products & Drug Development, Kaohsiung Medical University, Kaohsiung, Taiwan; ^10^National Institute of Allergy and Infectious Diseases, National Institutes of Health, Bethesda, MD, United States; ^11^School of Medical Laboratory Science and Biotechnology, College of Medical Science and Technology, Taipei Medical University, Taipei, Taiwan; ^12^Department of Cosmetic Science, Providence University, Taichung, Taiwan; ^13^Graduate Institute of Integrated Medicine, School of Chinese Medicine, China Medical University, Taichung, Taiwan

**Keywords:** HIV-1, antiretroviral drug, lipodystrophy, metabolic syndrome, neurologic function

## Abstract

Antiretroviral (ART) drugs has previously been associated with lipodystrophic syndrome, metabolic consequences, and neuropsychiatric complications. ART drugs include three main classes of protease inhibitors (PIs), nucleoside analog reverse transcriptase inhibitors (NRTIs), and non-nucleoside reverse transcriptase inhibitors (NNRTIs). Our previous work demonstrated that a high risk of hyperlipidemia was observed in HIV-1-infected patients who received ART drugs in Taiwan. Patients receiving ART drugs containing either Abacavir/Lamivudine (Aba/Lam; NRTI/NRTI), Lamivudine/Zidovudine (Lam/Zido; NRTI/NRTI), or Lopinavir/Ritonavir (Lop/Rit; PI) have the highest risk of hyperlipidemia. The aim of this study was to investigate the effects of Aba/Lam (NRTI/NRTI), Lam/Zido (NRTI/NRTI), and Lop/Rit (PI) on metabolic and neurologic functions in mice. Groups of C57BL/6 mice were administered Aba/Lam, Lam/Zido, or Lop/Rit, orally, once daily for a period of 4 weeks. The mice were then extensively tested for metabolic and neurologic parameters. In addition, the effect of Aba/Lam, Lam/Zido, and Lop/Rit on lipid metabolism was assessed in HepG2 hepatocytes and during the 3T3-L1 preadipocyte differentiation. Administration with Aba/Lam caused cognitive and motor impairments in mice, as well as their metabolic imbalances, including alterations in leptin serum levels. Administration with Lop/Rit also caused cognitive and motor impairments in mice, as well as their metabolic imbalances, including alterations in serum levels of total cholesterol, and HDL-c. Treatment of mice with Aba/Lam and Lop/Rit enhanced the lipid accumulation in the liver, and the decrease in AMP-activated protein kinase (AMPK) phosphorylation and/or its downstream target acetyl-CoA carboxylase (ACC) protein expression. In HepG2 hepatocytes, Aba/Lam, Lam/Zido, and Lop/Rit also enhanced the lipid accumulation and decreased phosphorylated AMPK and ACC proteins. In 3T3-L1 pre-adipocyte differentiation, Aba/Lam and Lop/Rit reduced adipogenesis by decreasing expression of transcription factor CEBPb, implicating the lipodystrophic syndrome. Our results demonstrate that daily oral administration of Aba/Lam and Lop/Rit may produce cognitive, motor, and metabolic impairments in mice, regardless of HIV-1 infection.

## Introduction

With the current increasing use of a combination of antiretroviral (ART) drugs, HIV-1/AIDS has become a treatable, chronic disease and patients can now expect improved overall health and life span (Deeks et al., 2013). ART drugs include three main classes of protease inhibitors (PIs), nucleoside analog reverse transcriptase inhibitors (NRTIs), and/or non-nucleoside reverse transcriptase inhibitors (NNRTIs). However, ART treatment of HIV-1-infected patients has been associated with an increased risk of metabolic consequences, lipodystrophy syndrome, and neuropsychiatric complications (Jevtovic et al., 2009; Paula et al., 2013; Finkelstein et al., 2015; Pedrol et al., 2015). ART treatment cause metabolic consequences such as abnormal glucose metabolism, insulin resistance, dyslipidemia, fatty liver, therefore resulting in type 2 diabetes, cardiovascular diseases, and stroke (Paula et al., 2013).

The cumulative use, adherence, and treatment regimen of ART drugs used may affect the risk of development of hyperlipidemia in HIV-1-infected patients (Jones et al., 2005; Anastos et al., 2007; Tsai et al., 2017). Signaling of the AMP-activated protein kinase (AMPK) protein and its downstream target, acetyl-coenzyme A carboxylase protein, are involved in lipid metabolism, and may contribute to hepatic tissue lipids and hyperlipidemia (Zang et al., 2004, 2006; Hou et al., 2008). In addition, adipogenic differentiation markers and transcription factors including fatty acid binding protein 4 (FABP4), adiponectin (Adipoq), peroxisome proliferator-activated receptor-gamma (PPARg), lipoprotein lipase (Lpl), sterol regulatory element-binding protein-1 (SREBP-1), CCAAT enhancer-binding proteins (CEBPs), and fatty acid synthetase (FAS) in adipose tissues may contribute to adipogenesis (Kim et al., 1998; Rosen and Spiegelman, 2000; Gougeon et al., 2004; Chen et al., 2017). Several drugs including thiazolidinediones and leptins have ameliorated symptoms in patients on ART (Mulligan et al., 2009; Edgeworth et al., 2013; Tsoukas et al., 2015). Thiazolidinediones are a class of PPAR agonists, that upregulate PPAR-dependent genes such as adiponectin, and showed a beneficial effect on limb fat mass in HIV-1/ART-associated lipodystrophy syndrome patients (Edgeworth et al., 2013). Furthermore, leptin expression has been shown to be regulated by several transcriptional factors including CEBPb (Mason et al., 1998). Leptin therapy has been found to improve hyperlipidemia and lipodystrophy in HIV-1-lipodystrophic patients (Mulligan et al., 2009; Tsoukas et al., 2015). Furthermore, leptin may have important physiological effects on brain function (Harvey, 2007; Chen et al., 2009). Leptins are known to have a significant effect on the hippocampus, a key brain region for cognition, and therefore may play important roles in neurologic complications (McGregor and Harvey, 2017a,b). However, further investigation is needed into the effects of these ART drugs and their action mechanisms.

Due to the complicated ART drug combinations involved in ART treatment and the lack of an appropriate animal experimental model, determining which PIs, NRTIs, and NNRTIs may be the main cause of ART-associated lipodystrophic syndrome, metabolic consequences, and neuropsychiatric complications is a complex task. Our study demonstrates that Taiwanese HIV-1-infected patients who received an NRTI/NRTI-containing regimen had the highest hyperlipidemia risk, followed by patients receiving a PI-containing regimen (Tsai et al., 2017). Amongst these NRTI/NRTI-containing regimens, the abacavir/lamivudine (Aba/Lam) and lamivudine/zidovudine (Lam/Zido) regimens were associated with the highest risk of hyperlipidemia. Amongst the PI-containing regimens, the lopinavir/ritonavir (Lop/Rit) regimen was associated with the highest risk of hyperlipidemia.

As previous work from our group demonstrated that a high risk of hyperlipidemia was observed in HIV-1-infected patients who received ART regimens in Taiwan, further studies are needed to investigate the most commonly used ART regimens to better understand their effect and possible mechanisms. Therefore, this study was designed to determine the effect of ART drugs on the development of metabolic consequences, lipodystrophy syndrome, cognitive and motor abilities in C57BL/6 mice. Mice were administered Aba/Lam, Lam/Zido, or Lop/Rit, orally, once daily for a period of 4 weeks. Here, we show that Aba/Lam and Lop/Rit regimens may contribute to the development of neurological and metabolic impairments in mice, suggesting that they are also likely to contribute to metabolic consequences, lipodystrophy syndrome, and neurologic complications in humans.

## Materials and Methods

### Animals

Six- to eight-week-old male C57Bl/6 mice were purchased from the National Laboratory Animal Center in Taiwan (AAALAC approved institute for animal propagation). All mice were housed in standard caging in temperature and humidity-controlled rooms with 12:12-h light:dark cycle and ad libitum access to dry laboratory food and water. Cages were cleaned once weekly. The use of mice our experiments complied with the 3R guidelines (3R: Replace, reduce, and refine) for the use of experimental animals. This study was reviewed and approved for all experimental protocols by the Institutional Animal Care and Use Committee (IACUC) in China Medical University, Taichung, Taiwan.

### Study Design

C57Bl/6 mice (6- to 8-week-old) were administered either 150/75 mg/kg Aba/Lam (Kivexa, Abbott Laboratories), 150/75 mg/kg Lam/Zido (Combivir, ViiV Healthcare, GlaxoSmithKline plc), or 150/37.5 mg/kg Lop/Rit (Kaletra, Abbott Laboratories), daily, via the oral route. The drugs were diluted in a control solution of 10% ethanol/15% propylene glycol. The control group of mice received only a solution of 10% ethanol/15% propylene via oral gavage with 100 μl. The dosage conversion was based on body surface area (BSA) normalization factors, which translate from 10 mg/kg in humans to approximately 125 mg/kg in mice (Reagan-Shaw et al., 2008). Following a period of 4 weeks for daily treatment, groups of mice were sacrificed by CO_2_ asphyxiation (*n* = 4 per treatment group per time; repeated twice).

**FIGURE 1:**
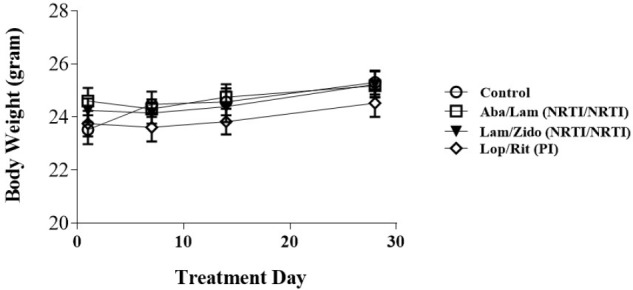
Effects of antiretroviral drugs on body weight in mice. Male C57BL/6 mice were orally administrated daily with control (open circles), Aba/Lam (abacavir/lamivudine (NRTI/NRTI); open squares), Lam/Zido (lamivudine/zidovudine (NRTI/NRTI); closed triangles), and Lop/Rit (Lopinavir/ritonavir (PI); open diamond). These four groups were evaluated daily for a period of 4 weeks for their body weight (gram). All data are mean ± SEM for each group (8–10 mice per group). Data are analyzed by the un-paired student *t*-test in the experimental groups Aba/Lam, Lam/Zido, or Lop/Rit-treated mice as compared with the control mice at the same time point.

**FIGURE 2:**
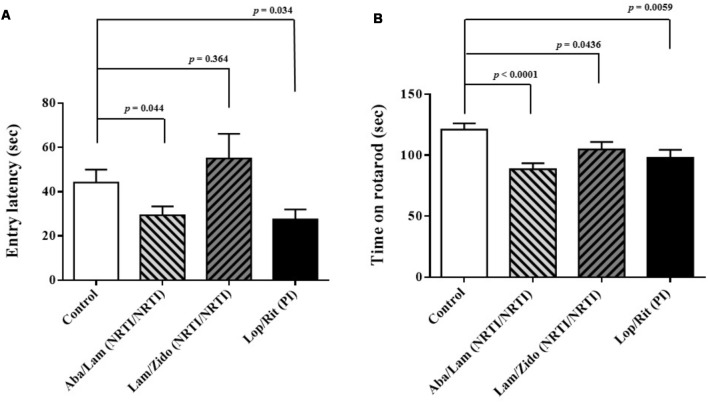
Effects of antiretroviral drugs (Aba/Lam, Lam/Zido, and Lop/Rit) on cognitive and motor abilities in the C57Bl/6 mice. **(A)** Effects of antiretroviral drugs on cognitive performance in the passive avoidance test. **(B)** Effects of antiretroviral drugs on motor performance in the rotarod test. All data are mean ± SEM for each group (8–10 mice per group). Data are expressed as the time (sec) and analyzed by the un-paired student *t*-test in the experimental groups Aba/Lam, Lam/Zido, or Lop/Rit-treated mice as compared with the control mice.

Body weight was measured daily for a period of 4 weeks (**Figure 1**). Passive avoidance tests (cognitive performance) were performed at day 29 (the Gemini Avoidance System, San Diego Instruments, San Diego, CA, United States; **Figure 2**). Briefly, mice were placed in the right chamber (bright room). After 5 s, the light in the right chamber was turned on, and the gate was opened simultaneously. Once the mice entered the left chamber (dark room), the gate was then quickly closed, and the entry latency (sec) was recorded. Each trial was repeated three times. In addition, a timed rotarod test (motor performance) was also performed at day 30 (Rotamex, Columbus Instrument, OH, United States; **Figure 2**). Briefly, mice were placed on a Rotamex with an initial speed of 4 rpm. The speed of the Rotamex was increased by 0.5 rpm per 5 s until the maximum 20 rpm/min speed was reached. Each mouse was tested on six separate occasions.

**FIGURE 3:**
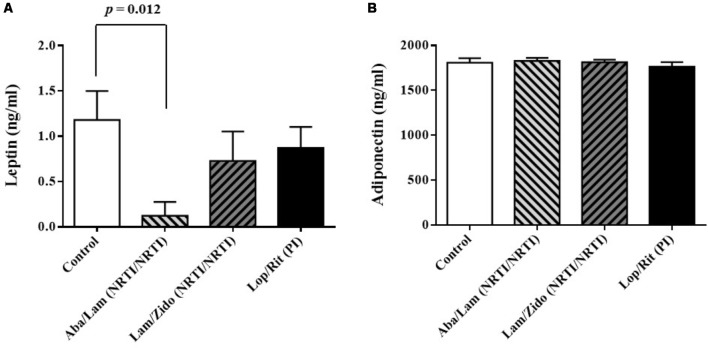
Effects of antiretroviral drugs (Aba/Lam, Lam/Zido, and Lop/Rit) on serum levels of adipokines in the C57Bl/6 mice. **(A)** Effects of antiretroviral drugs on serum levels of leptin. **(B)** Effects of antiretroviral drugs on serum levels of adiponectin. All data are mean ± SEM for each group (8–10 mice per group). Data are analyzed by the un-paired student *t*-test in the experimental groups Aba/Lam, Lam/Zido, or Lop/Rit-treated mice as compared with the control mice.

**FIGURE 4:**
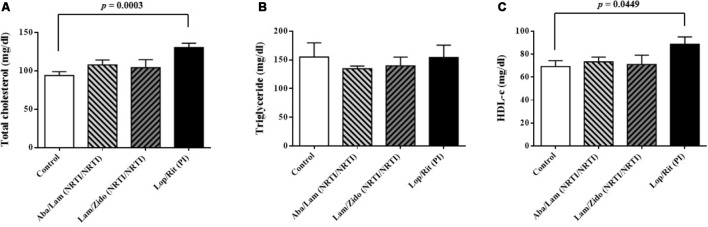
Effects of antiretroviral drugs (Aba/Lam, Lam/Zido, and Lop/Rit) on serum lipid levels in the C57Bl/6 mice. **(A)** Effects of antiretroviral drugs on serum level of total cholesterol. **(B)** Effects of antiretroviral drugs on serum level of triglyceride. **(C)** Effects of antiretroviral drugs on serum level of HDL-c. All data are mean ± SEM for each group (8–10 mice per group). Data are analyzed by the un-paired student *t*-test in the experimental groups Aba/Lam, Lam/Zido, or Lop/Rit-treated mice as compared with the control mice at the same time point.

Blood was collected by cardiac puncture of terminally anesthetized mice at day 31. Clotted blood was then centrifuged at 3,000 × *g* for 30 min in order to obtain serum. The serum levels of leptin (**Figure 3**; EML2001-1; Mouse Leptin ELISA Kit) and adiponectin (**Figure 3**; EMA2500-1; Mouse Adiponectin ELISA Kit) were also evaluated by ELISA according to the manufacturer’s protocol (Assaypro, MO, United States). The serum levels of total cholesterol, HDL-cholesterol, and triglycerides were measured colorimetrically using commercially available kits (**Figure 4**; SPOTCHEM EZ SP-4430, ARKRAY, Yu-Shing Biotech., Ltd, Taipei, Taiwan).

**FIGURE 5:**
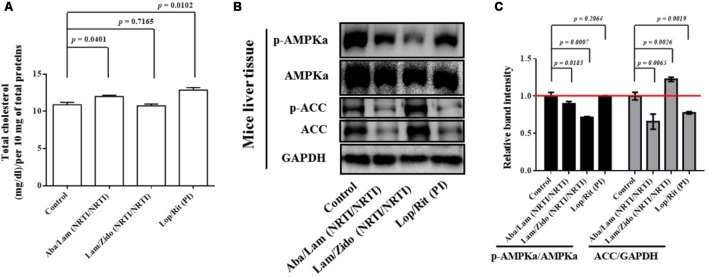
Effects of antiretroviral drugs (Aba/Lam, Lam/Zido, and Lop/Rit) on total cholesterol content, and lipid metabolism-related AMPK and ACC proteins in the liver tissue of C57Bl/6 mice. **(A)** Total cholesterol levels of each group were detected in the liver tissue in mice and expressed in bar chart. **(B)** Effects of antiretroviral drugs (Aba/Lam, Lam/Zido, and Lop/Rit) on phosphorylation and expression of both AMPKa and ACC proteins by using Western blot analysis. **(C)** Relative ratios of band intensity of p-AMPKa/AMPKa and ACC/GAPDH when compared with the controls. Western blot band intensities were quantified by using Image J software. All data are mean ± SEM for each group (8–10 mice per group). Data are analyzed by the un-paired student *t*-test in the experimental groups Aba/Lam, Lam/Zido, or Lop/Rit-treated mice as compared with the control mice at the same time point.

Liver tissue samples were collected from euthanized mice for Western blot analysis (**Figure 5**). Briefly, liver tissue samples were homogenized on ice with the RIPA protein extraction kit (Pierce, Thermo Fisher Scientific, Rockford, IL, United States) with a PI (complete EDTA-freePI, Roche Life Science, Sigma-Aldrich) according to the manufacturer’s protocol. The cell lysates were centrifuged to remove insoluble protein. The protein concentrations of the supernatants of cell lysates were determined using the BCA protein assay kit. Samples of cell lysates with equal protein concentrations were subjected to 12% SDS-PAGE and then transferred to polyvinylidene fluoride membranes (Millipore, Billerica, MA, United States). The membranes were then incubated with primary antibodies overnight at 4°C. The primary antibodies included anti-phospho-AMPKa (Thr172; 40H9, catalog number 2535), anti-AMPKa (D5A2, catalog number 5831), anti-phospho-Acetyl-CoA Carboxylase (Ser79; D7D11, catalog number 11818), and anti-Acetyl-CoA Carboxylase (C83B10, catalog number 3676) from Cell Signaling Technology, Inc. (Beverly, MA, United States), anti-actin (mAbGEa, catalog number NB100-74340) from Novus Biologicals, and anti-GAPDH (catalog number 60004-1-Ig) antibodies from Proteintech Group Inc. (Rosemont, IL, United States). The membranes were then incubated with alkaline phosphatase-conjugated secondary antibodies (Sigma-Aldrich). Signals were visualized using a chemiluminescence kit (Chemicon), following the manufacturer’s protocol. The Western blot band intensities were quantified by using Image J software. Phospho-AMPKa, total AMPKa, Phospho-ACC, total ACC, and GAPDH were all quantified from their appropriate protein mass bands on the blot. Relative ratios of band intensity of p-AMPKa/AMPKa and ACC/GAPDH were compared with the controls. All data are mean ± SEM for each group (8–10 mice per group). Data are analyzed by the un-paired student *t*-test in the experimental groups Aba/Lam, Lam/Zido, or Lop/Rit-treated mice as compared with the control mice at the same time point.

**FIGURE 6:**
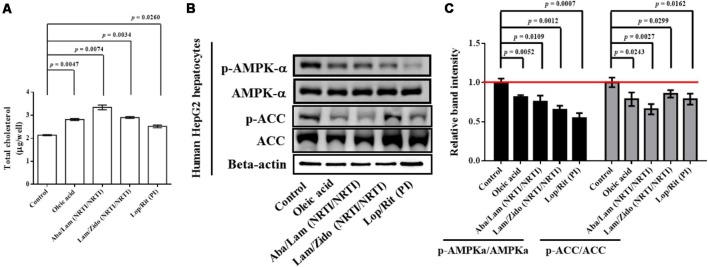
Effects of antiretroviral drugs (Aba/Lam, Lam/Zido, and Lop/Rit) on total cholesterol content, and lipid metabolism-related AMPK and ACC proteins in human HepG2 hepatocytes. **(A)** Total cholesterol levels of each group were assayed and expressed in bar charts. **(B)** Effects of antiretroviral drugs (Aba/Lam, Lam/Zido, and Lop/Rit) on phosphorylation and expression of both AMPKa and ACC proteins by using Western blot analysis. **(C)** Relative ratios of band intensity of p-AMPKa/AMPKa and p-ACC/ACC when compared with the controls. All data are mean ± SEM for each group. Data are analyzed by the un-paired student *t*-test in the experimental groups Aba/Lam, Lam/Zido, or Lop/Rit-treated HepG2 cells as compared with the control cells. Oleic acid-treated HepG2 cells are used as the lipid accumulation controls and the controls to inhibit the AMPK and ACC signaling pathway.

For HepG2 (human hepatocellular carcinoma) cell culture, the cells were maintained in Dulbecco’s modified Eagle’s medium supplemented with 10% fetal bovine serum, 100 U/mL penicillin, 100 U/mL streptomycin, and 2 mM L-glutamine (Gibco, Thermo Fisher Scientific, Waltham, MA, United States). Human HepG2 cells were treated with ART drugs for 24 h at the concentrations indicated (**Figure 6**). The cells were then lysed in RIPA buffer and the proteins resolved by SDS-PAGE and western blot analysis as previously described for mouse liver tissue samples (**Figure 5**). Western blot band intensities were quantified using ImageJ, as described above. Oleic acid-treated HepG2 cells are used as the lipid accumulation controls and the controls to inhibit the AMPK and ACC signaling pathway.

**FIGURE 7:**
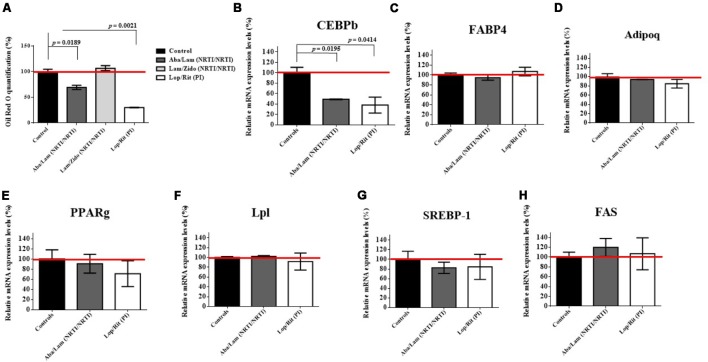
Effects of antiretroviral drugs (Aba/Lam, Lam/Zido, and Lop/Rit) on mouse 3T3-L1 preadipocytes differentiation. Mouse 3T3-L1 preadipocytes were differentiated in differentiation medium in the presence of the indicated concentrations of antiretroviral drugs for 7 days. **(A)** Oil Red O quantification analysis of antiretroviral drugs in 3T3-L1 preadipocytes differentiation under adipocyte differentiation medium. Effect of antiretroviral drugs on the relative mRNA expression levels for **(B)** CEBPb **(C)** FABP4, **(D)** adipoq, **(E)** PPARg, **(F)** Lpl, **(G)** SREBP-1, and **(H)** FAS when compared with the controls by using RT-qPCR analysis. All data are mean ± SEM for each group for three independent experiments. Data are analyzed by the un-paired student *t*-test in the experimental groups Aba/Lam, Lam/Zido, or Lop/Rit-treated cells as compared with the controls.

For 3T3-L1 (mouse preadipocyte) cell culture, the cells were maintained in Dulbecco’s modified Eagle’s medium as previously described. Mouse 3T3-L1 preadipocytes were differentiated in differentiation medium in the presence of ART drugs for 7 days at the concentrations indicated (**Figure 7**). Oil Red O quantification analysis of ART drugs in 3T3-L1 preadipocytes differentiation under adipocyte differentiation medium (Cat No.: DIF001, 3T3-L1 Differentiation Kit, Sigma-Aldrich, Inc.) was performed by using Lipid (Oil Red O) Staining Kit (Cat No.: K580-24, BioVision).

Cellular RNA was isolated from cells treated with ART drugs using a QIAamp^®^ RNA Mini Kit according to the manufacturer’s instructions (Qiagen, Valencia, CA, United States). RNAs were eluted in 60 μL buffer. For Roche probe and primer hybridization assays (Universal ProbeLibrary System Assay Design, Roche), the primers used for quantitative PCR (qPCR) amplification were found by using ProbeFinder (a web-based software tool) as follows: *CEBP-b*, forward: 5’-tgatgcaatccggatcaa-3’ and reverse: 5’-cacgtgtgttgcgtcagtc-3’ (UPL probe number 102). Reverse transcription and Real-time RT-PCR was performed as previously described (Lin et al., 2015). The genomic DNAs were removed by RNase-Free DNase Set (Cat No.: 79254, Qiagen) and the primers designed for qPCR were all spanned across an exon-exon junction as described by Roche probe and primer hybridization assays.

### Statistical Analyses

All data were calculated as mean ± SEM for each group for three independent experiments using GraphPrism software (GraphPad Software, Inc., La Jolla, CA, United States). All data were analyzed using two-tailed, unpaired student *t*-tests to determine statistical differences in mice, HepG2 cells, and 3T3-L1 cells. All *p*-values of less than 0.05 were considered to be statistically significant.

## Results

### Effects of ART Drugs (Aba/Lam, Lam/Zido, and Lop/Rit) on Body Weight in C57Bl/6 Mice

Patients who received nucleoside reverse-transcriptase inhibitors (NRTI/NRTI)-containing regimens were found to have the highest hyperlipidemia risk, followed by those receiving PI- containing and non-NRTI-containing regimens (Tsai et al., 2017). The current study was undertaken to determine the effects of ART drug regimens Aba/Lam, Lam/Zido, and Lop/Rit, on both metabolic and cognitive function in C57Bl/6 mice. Six- to eight-week-old male C57Bl/6 mice were administered with either control (the mixture of 10% ethanol/15% propylene) or Aba/Lam (150/75 mg/kg) (Kivexa, Abbott Laboratories), or Lam/Zido (150/75 mg/kg) (Combivir, ViiV Healthcare, GlaxoSmithKline plc), or Lop/Rit (150/37.5 mg/kg) (Kaletra, Abbott Laboratories), daily via the oral route for a period of 4 weeks. The body weight of the mice was recorded regularly as a health index. As shown in **Figure 1**, the treated mice showed no significant differences from the control mice. These data suggest that the drugs had no significant effect on body weight.

### Effects of ART Drugs (Aba/Lam, Lam/Zido, and Lop/Rit) on Cognitive and Motor Abilities in C57Bl/6 Mice

To investigate the effects of ART drugs (Aba/Lam, Lam/Zido, and Lop/Rit) on cognitive and motor abilities, passive avoidance and rotarod tests were used for behavioral analyses. The passive avoidance test was used for cognitive function evaluation following 4 weeks of ART drug exposure in this study. The passive avoidance test is a fear-aggravated test that is used to evaluate learning and memory in rodents with central nervous system (CNS) disorders^1^ and is also useful for evaluating the effect of exposure to chemicals on learning and memory as well as for studying the mechanisms involved in cognition (Beheshti et al., 2016). By recording the entry latency (sec), we observed that Aba/Lam and Lop/Rit treatments resulted in cognitive impairment (**Figure 2A**; *p* = 0.044 for the Aba/Lam treated group; *p* = 0.034 for the Lop/Rit treated group). In addition, the rotarod test is a very commonly used test for evaluating motor performance in mice. The time remaining on rotarod was measured when the mice were on an accelerating rotating cylinder. As shown in **Figure 2B**, there were significant differences between these groups. By recording the time on rotarod when accelerating the rotating cylinder, we observed that treatment of mice with Aba/Lam, Lam/Zido, or Lop/Rit did lead to motor impairment (**Figure 2B**; *p* < 0.0001 for the Aba/Lam treated group; *p* = 0.0436 for the Lam/Zido treated group; *p* = 0.0059 for the Lop/Rit treated group).

### Effects of ART Drugs (Aba/Lam, Lam/Zido, and Lop/Rit) on Metabolic Parameters in C57Bl/6 Mice

Following a period of 4 weeks for daily treatment regimen of either control or abacavir/lamivudine, or lamivudine/zidovudine, or lopinavir/ritonavir administered orally, the blood samples were collected to obtain their serum. The serum levels of leptin, adiponectin, total cholesterol, HDL-cholesterol, and triglycerides from these groups were measured (**Figures 3**, **4**). As shown in **Figure 3A**, there were significant differences in serum levels of leptin. Lower serum levels of leptin were observed in mice treated with Aba/Lam than in the controls (*p* = 0.012 for the Aba/Lam treated group). However, there was no significant difference in the serum level of adiponectin between these groups (**Figure 3B**).

Serum samples were obtained following treatment with ART drugs as described. As shown in **Figure 4A**, increased serum levels of total cholesterol were observed in mice treated with Lop/Rit (*p* = 0.0003 for the Lop/Rit treated group). There were no significant differences in the serum levels of triglyceride (**Figure 4B**). Furthermore, increased serum levels of HDL-c were observed in mice treated with Lop/Rit (**Figure 4C**; *p* = 0.0449 for the Lop/Rit treated group). These results suggest that mice treated with Lop/Rit, may show increased serum levels of total cholesterol and HDL-c, respectively.

Studies have reported that the signaling mechanism involved in the phosphorylation and expression of AMPK protein and its downstream target, ACC protein, results in fatty acid oxidation and lipid synthesis, two important lipid metabolism-related proteins of hepatic tissue lipids and hyperlipidemia (Zang et al., 2004, 2006; Hou et al., 2008). Phosphorylation and expression of both AMPKa and ACC proteins in mice were also detected using western blot analysis.

As shown in **Figure 5A**, the Aba/Lam and Lop/Rit regimens were found to significantly increase intracellular lipid accumulation in comparison to that observed in the controls in the total cholesterol quantification assay (*p* = 0.0401 for the Aba/Lam treated group; *p* = 0.0102 for the Lop/Rit treated group). As shown in **Figures 5B,C**, the phosphorylation of AMPKa protein decreased in groups treated with Aba/Lam and Lam/Zido when compared with the control level (*p* = 0.0183 for the Aba/Lam treated group; *p* = 0.0007 for the Lam/Zido treated group). The protein expression of ACC significantly decreased in the groups treated with Aba/Lam and Lop/Rit in comparison with the control level (*p* = 0.0065 for the Aba/Lam treated group; *p* = 0.0019 for the Lop/Rit treated group).

### Effects of ART Drugs (Aba/Lam, Lam/Zido, and Lop/Rit) on the Lipid Accumulation, Phosphorylation and Expression of Both AMPKa and ACC Proteins in Human HepG2 Hepatocytes

To determine whether ART drugs affect lipogenesis, hepG2 hepatocytes were treated with Aba/Lam, Lam/Zido, or Lop/Rit. Oleic acid-treated HepG2 cells are used as the lipid accumulation controls and the controls to inhibit the AMPK and ACC signaling pathway. Total cholesterol levels of each group were assayed and expressed in bar charts (**Figure 6A**). As shown, treatment with oleic acid, Aba/Lam, Lam/Zido, and Lop/Rit led to intracellular total cholesterol accumulation (*p* = 0.0047 for the oleic acid treated group; *p* = 0.0074 for the Aba/Lam treated group; *p* = 0.0034 for the Lam/Zido treated group; *p* = 0.0260 for the Lop/Rit treated group). The phosphorylation and expression of the AMPKa and ACC proteins were subsequently determined using western blot analysis. As shown in **Figures 6A,B**, treatment with oleic acid, Aba/Lam, Lam/Zido, and Lop/Rit led to decreased phosphorylation of the AMPK protein (*p* = 0.0052 for the oleic acid treated group; *p* = 0.0109 for the Aba/Lam treated group; *p* = 0.0012 for the Lam/Zido treated group; *p* = 0.0007 for the Lop/Rit treated group). Similarly, treatment with oleic acid, Aba/Lam, Lam/Zido, and Lop/Rit led to decreased phosphorylation of the ACC protein (*p* = 0.0243 for the oleic acid treated group; *p* = 0.0027 for the Aba/Lam treated group; *p* = 0.0299 for the Lam/Zido treated group; *p* = 0.0162 for the Lop/Rit treated group).

### Effects of ART Drugs (Aba/Lam, Lam/Zido, and Lop/Rit) on Lipid Accumulation and Adipocyte Differentiation Marker in 3T3-L1 Pre-adipocytes

Long term ART use has been associated with lipodystrophic syndrome, which causes alterations in body fat distribution and lipid metabolism (Miller et al., 1998; Stocker et al., 1998). To evaluate the effects of Aba/Lam, Lam/Zido, and Lop/Rit on pre-adipocyte differentiation, 3T3-L1 preadipocytes were cultured for 7 days in the presence of ART drugs at the concentrations indicated (**Figure 7**). As shown in **Figure 7A**, the Aba/Lam and Lop/Rit regimens were found to significantly decrease intracellular lipid accumulation in comparison to that observed in the controls in the Oil red O quantification assay (*p* = 0.0189 for the Aba/Lam treated group; *p* = 0.0021 for the Lop/Rit treated group). However, no significant differences in lipid accumulation were observed between Lam/Zido-treated cells and the controls. These results suggest that exposure to either Aba/Lam or Lop/Rit during pre-adipocyte differentiation significantly reduces lipid accumulation.

Aba/Lam and Lop/Rit significantly reduced lipid accumulation in differentiating adipocytes. Several adipogenic differentiation markers and transcription factors are involved in the molecular mechanism of adipogenesis (Kim et al., 1998; Rosen and Spiegelman, 2000; Gougeon et al., 2004; Chen et al., 2017). In this study, we examined the expression of adipogenic differentiation markers and transcription factors by incubating 3T3-L1 pre-adipocytes with either Aba/Lam or Lop/Rit for 7 days using RT-qPCR analysis (**Figure 7**). These adipogenic differentiation markers and transcription factors include FABP4, adipoq, PPARg, Lpl, SREBP-1, CEBPb, and FAS proteins. As shown, there were no significant differences in these adipogenic differentiation markers and transcription factors, except for the CEBPb protein. 3T3-L1 pre-adipocytes treated with Aba/Lam or Lop/Rit, showed the most significant inhibition of CEBPb expression (**Figure 7B**; *p* = 0.0195 for the Aba/Lam treated group; *p* = 0.0414 for the Lop/Rit treated group). These results suggest that the Aba/Lam and Lop/Rit combinations may inhibit lipid accumulation in differentiating adipocytes by down-regulating CEBPb adipogenic transcription factor expression.

## Discussion

This study was initiated based on our previous longitudinal, comprehensive, and population-based study performed in Taiwan, which demonstrated that cumulative ART use, ART adherence, and the type of regimen provided may increase hyperlipidemia risk in HIV-1-infected patients when compared to HIV-1-infected patients who did not receive ART treatment (Tsai et al., 2017). A high risk of hyperlipidemia was observed in HIV-1-infected patients who received Aba/Lam, Lam/Zido, or Lop/Rit drug regimens. Therefore, in this study, we observed that daily oral administration of Aba/Lam or Lop/Rit for 4 weeks in mice can contribute to cognitive, motor, and metabolic impairment. Our results demonstrate that daily oral administration of Aba/Lam or Lop/Rit may produce cognitive, motor, and metabolic impairments in mice, regardless of HIV-1 infection.

Our results demonstrate that mice treated with either the Aba/Lam or Lop/Rit drug regimens exhibit cognitive impairment. Motor impairment was observed in mice treated with Aba/Lam, Lam/Zido, and Lop/Rit. These results suggest that in addition to HIV-1 infection, these ART drugs may also affect neurologic functions. Further, to investigate whether a correlation exists between HIV-1-infected patients receiving ART and neurological disorders, we performed a longitudinal and population-based study to investigate the effect of different ART regimens on the risk of neurological disorders in the Taiwanese HIV-1/ART cohort (**Supplementary Tables S1**, **S2**). We found that patients receiving nucleoside reverse-transcriptase inhibitors (NRTI/NRTI)-containing regimen had the risk of neurological disorders. Patients receiving PI-containing regimens had the risk of neurological disorders. HIV-1-associated neuropsychiatric disorders (HAND) are mainly due to HIV-1 infection and AIDS progression and remain a significant public health concern (Cespedes and Aberg, 2006; Pedrol et al., 2015). HAND prevalence remains high even in the post-ART era (Heaton et al., 2010; Ciccarelli et al., 2011; Carvalhal et al., 2016). Although ART is known to protect the CNS against HIV-1 infection and prevent the development of local HIV-1 reservoirs, the effectiveness of CNS penetration by these ART drugs and their potential neurotoxicities need further study (Ciccarelli et al., 2011; Fabbiani et al., 2015; Stauch et al., 2017). Efavirenz is the ART drug that has been the most extensively studied in relation to HAND. Efavirenz has been associated with HAND in both human clinical studies and murine models (Romao et al., 2011; Streck et al., 2011; Ma et al., 2016) and has been shown to affect brain energy metabolism dysfunction, especially in the cerebral cortex, striatum, and hippocampus (Streck et al., 2011). In addition, Efavirenz promotes beta-secretase (BACE-1) expression and increases Aβ generation in SweAPP N2a neural cells and in a mouse model (Brown et al., 2014). However, Efavirenz lowered the clearance of Aβ peptide in primary microglial phagocytosis cells (Brown et al., 2014). Furthermore, Efavirenz reduced neural stem cell proliferation in rat NSC cells and in a mouse model (Jin et al., 2016). Lop/Rit led to poor performance in neurocognitive functioning in both HIV-1-infected patients (Ma et al., 2016) and mouse models (Pistell et al., 2010; Gupta et al., 2012). In addition, both Aba/Lam and Lop/Rit induced an increase in Aβ generation in SweAPP N2a neural cells and reduced the clearance of Aβ peptide in N9 microglial phagocytosis cells (Giunta et al., 2011), suggesting that these ART drugs may contribute to the cognitive and motor decline observed in HIV-1-infected patients.

Our recent study demonstrated that factors including cumulative use, adherence, and the treatment regimen of ART given, may affect metabolic function, and particularly hyperlipidemia risk, in HIV-1-infected patients in a dose-dependent manner (Tsai et al., 2017). HIV-1-infected patients receiving Aba/Lam-containing regimens were also found to have increased blood cholesterol levels (Moyle et al., 2015). Lop/Rit has been also associated with hyperlipidemia in both human clinical studies and murine models (Gupta et al., 2012; Wangpatharawanit and Sungkanuparph, 2016; Matoga et al., 2017). Our results also showed that treatment of mice with Aba/Lam and Lop/Rit enhanced the lipid accumulation in the liver, and decreased AMPK phosphorylation and/or the expression of ACC acetyl-CoA. ART drugs may bind to the LDL receptor-related protein (LRP), thereby inhibiting the function of the LRP-Lpl complex (the cleavage of fatty acids from plasma triglycerides) (Carr et al., 1998; Matoga et al., 2017). ART drugs may also induce abnormal lipid metabolism by causing alterations in particular genes, including genes encoding sterol regulatory element-binding proteins, cytoplasmic retinoic-acid binding proteins, peroxisome proliferator activated receptors, and apoCIII (Prot et al., 2006; Matoga et al., 2017). Furthermore, tissue lipids and hyperlipidemia may be affected by the signaling mechanism that is involved in phosphorylation and expression of AMPK protein and its downstream target, ACC protein, which is known to play a role in fatty acid oxidation and lipid synthesis (Zang et al., 2004, 2006; Hou et al., 2008). The reduced signaling in AMPK and ACC proteins contributed to the impaired fatty acid oxidation and then resulted in lipid accumulation in liver tissue (Zang et al., 2004, 2006; Hou et al., 2008). Our results also demonstrate that Aba/Lam, Lam/Zido, and Lop/Rit led to intracellular total cholesterol accumulation and decreased the phosphorylation of AMPK and ACC proteins *in vitro* HepG2 cells. Our results also showed that these ART drugs under AMPK antagonist dorsomorphin impaired the AMPK and ACC signaling pathway (**Supplementary Figure S1**). The treatment of mice with AMPK antagonist dorsomorphin alone or combined with ART drugs will be investigated in our next study.

Our results demonstrate that Aba/Lam and Lop/Rit attenuate 3T3-L1 pre-adipocyte differentiation by inhibiting CEBPb adipogenic transcription factor expression, implicating the lipodystrophic syndrome. In addition, leptin serum levels were found to be reduced in mice treated with Aba/Lam. These results suggest that these ART drugs may affect body fat distribution and lipid metabolism (Miller et al., 1998; Stocker et al., 1998). Indeed, long-term use of ART has been associated with lipodystrophic syndrome (Finkelstein et al., 2015). Decreased levels of leptin have previously been observed in HIV-1-lipodystrophic patients (Tiliscan et al., 2015; Tsoukas et al., 2015). This decrease inversely correlates with the occurrence of hyperlipidemia (Nagy et al., 2003). Leptin expression has been shown to be regulated by several transcriptional factors including CEBPb (Mason et al., 1998). Leptin therapy in HIV-1-lipodystrophic patients has been found to improve blood lipid profiles, and to decrease visceral fat without having any effect on subcutaneous or peripheral fat distribution (Mulligan et al., 2009; Tsoukas et al., 2015). Furthermore, leptins are known to have a significant effect on the hippocampus, a key brain region for cognition, and therefore may play important roles in neurological complications (McGregor and Harvey, 2017a,b).

Several ART drugs are associated with HIV-1-associated lipodystrophy (Zhang et al., 1999; Caron et al., 2004). Ritonavir has previously been associated with reduced triglyceride accumulation and was found to inhibit aP2, Lpl, and Adipoq adipogenic differentiation markers in 3T3-L1 pre-adipocyte differentiation (Zhang et al., 1999). Zidovudine was found to reduce lipid content in differentiated 3T3-L1 pre-adipocytes by inhibiting FAS, ACC, and aP2 adipogenic markers (Caron et al., 2004). Lam/Zido and Aba/Lam/Zido also reduced lipid content in differentiated 3T3-L1 preadipocytes (Caron et al., 2004). Despite the beneficial effects on lipdystrophy observed in studies using thiazolidinediones (Edgeworth et al., 2013), our results showed that PPARg was significantly reduced and thus may not be a druggable target. Our study demonstrates that Aba/Lam and Lop/Rit also reduce lipid accumulation in adipocytes by inhibiting CEBPb adipogenic transcription factor expression. Furthermore, serum leptin levels were found to be reduced in Aba/Lam-treated mice.

## Conclusion

In this study, there is a limitation of applicability of the mice cognitive function tests to human neurological disorders. The cognitive function tests used in mice are not absolutely relevant for the determining the neurological consequences in human. However, this study demonstrated that daily, oral administration of Aba/Lam and Lop/Rit may increase the likelihood of the development of cognitive, motor, and metabolic impairments in mice, regardless of HIV-1 infection. The AMPK-ACC signaling, CEBPb adipogenic transcription factor, and leptin may play roles in the molecular mechanisms of lipid metabolism and neuroprotection. Further studies are required to devise treatments that reduce or avoid the development of ART-associated metabolic and neurologic complications.

## Author Contributions

F-JT, M-WH, C-HL, W-ML, and Y-JL conceived and designed the experiments. C-HC, J-PL, C-FC, T-HL, C-CLiao, and S-MH performed the experiments. C-FC analyzed the data. Y-CW, XL, HT, J-CL, C-CLin, and C-LH contributed reagents, materials, and analysis tools. W-ML and Y-JL wrote the manuscript. All of the authors have read and approved the final manuscript.

## Conflict of Interest Statement

The authors declare that the research was conducted in the absence of any commercial or financial relationships that could be construed as a potential conflict of interest.

## Footnotes

1 https://med.stanford.edu/sbfnl/services/bm/lm/bml-passive.html

## Supplementary Material

FIGURE S1 Effects of antiretroviral drugs (Aba/Lam, Lam/Zido, and Lop/Rit) with dorsomorphin on lipid metabolism-related AMPK and ACC proteins in human HepG2 hepatocytes. **(A)** Effects of antiretroviral drugs (Aba/Lam, Lam/Zido, and Lop/Rit) with dorsomorphin on phosphorylation and expression of both AMPKa and ACC proteins by using Western blot analysis. **(B)** Relative ratios of band intensity of p-AMPKa/AMPKa and p-ACC/ACC when compared with the controls. All data are mean ± SEM for each group. Data are analyzed by the un-paired student *t*-test in the experimental groups Aba/Lam, Lam/Zido, or Lop/Rit-treated HepG2 cells as compared with the control cells. Dorsomorphin-treated HepG2 cells are used as the AMPK antagonist controls. Oleic acid-treated HepG2 cells are used as the controls to inhibit the AMPK and ACC signaling pathway.

TABLE S1 Demographic characteristics of HIV-1-infected patients with and without neurological disorders.

TABLE S2 Neurological disorder risk in HIV-1-infected patients according to single type of ART regimen.

## References

[B1] Anastos K. Lu D. Shi Q. Tien P. C. Kaplan R. C. Hessol N. A. (2007). Association of serum lipid levels with HIV serostatus, specific antiretroviral agents, and treatment regimens. *J. Acquir. Immune Defic. Syndr.* 45 34–42. 10.1097/QAI.0b013e318042d5fe 17460470

[B2] Beheshti F. Khazaei M. Hosseini M. (2016). Neuropharmacological effects of *Nigella sativa*. *Avicenna J. Phytomed.* 6 104–116. PMC4884225 27247928

[B3] Brown L. A. Jin J. Ferrell D. Sadic E. Obregon D. Smith A. J. (2014). Efavirenz promotes beta-secretase expression and increased Abeta_1_-40,42 via oxidative stress and reduced microglial phagocytosis: implications for HIV associated neurocognitive disorders (HAND). *PLoS One* 9:e95500. 10.1371/journal.pone.0095500 24759994 PMC3997351

[B4] Caron M. Auclair M. Lagathu C. Lombes A. Walker U. A. Kornprobst M. (2004). The HIV-1 nucleoside reverse transcriptase inhibitors stavudine and zidovudine alter adipocyte functions in vitro. *AIDS* 18 2127–2136. 10.1097/00002030-200411050-00004 15577645

[B5] Carr A. Samaras K. Chisholm D. J. Cooper D. A. (1998). Pathogenesis of HIV-1-protease inhibitor-associated peripheral lipodystrophy, hyperlipidaemia, and insulin resistance. *Lancet* 351 1881–1883. 10.1016/S0140-6736(98)03391-1 9652687

[B6] Carvalhal A. Gill M. J. Letendre S. L. Rachlis A. Bekele T. Raboud J. (2016). Central nervous system penetration effectiveness of antiretroviral drugs and neuropsychological impairment in the Ontario HIV Treatment Network Cohort Study. *J. Neurovirol.* 22 349–357. 10.1007/s13365-015-0404-5 26572786 PMC10748733

[B7] Cespedes M. S. Aberg J. A. (2006). Neuropsychiatric complications of antiretroviral therapy. *Drug Saf.* 29 865–874. 10.2165/00002018-200629100-00004 16970510

[B8] Chen B. Liao W. Q. Xu N. Xu H. Wen J. Y. Yu C. A. (2009). Adiponectin protects against cerebral ischemia-reperfusion injury through anti-inflammatory action. *Brain Res.* 1273 129–137. 10.1016/j.brainres.2009.04.002 19362080

[B9] Chen J. Liu Y. Lu S. Yin L. Zong C. Cui S. (2017). The role and possible mechanism of lncRNA U90926 in modulating 3T3-L1 preadipocyte differentiation. *Int. J. Obes.* 41 299–308. 10.1038/ijo.2016.189 27780975 PMC5309343

[B10] Ciccarelli N. Fabbiani M. Di Giambenedetto S. Fanti I. Baldonero E. Bracciale L. (2011). Efavirenz associated with cognitive disorders in otherwise asymptomatic HIV-infected patients. *Neurology* 76 1403–1409. break 10.1212/WNL.0b013e31821670fb 21502598

[B11] Deeks S. G. Lewin S. R. Havlir D. V. (2013). The end of AIDS: HIV infection as a chronic disease. *Lancet* 382 1525–1533. 10.1016/S0140-6736(13)61809-7 24152939 PMC4058441

[B12] Edgeworth A. Treacy M. P. Hurst T. P. (2013). Thiazolidinediones in the treatment of HIV/HAART-associated lipodystrophy syndrome. *AIDS Rev.* 15 171–180. 24002201

[B13] Fabbiani M. Grima P. Milanini B. Mondi A. Baldonero E. Ciccarelli N. (2015). Antiretroviral neuropenetration scores better correlate with cognitive performance of HIV-infected patients after accounting for drug susceptibility. *Antivir. Ther.* 20 441–447. 10.3851/IMP2926 25516553

[B14] Finkelstein J. L. Gala P. Rochford R. Glesby M. J. Mehta S. (2015). HIV/AIDS and lipodystrophy: implications for clinical management in resource-limited settings. *J. Int. AIDS Soc.* 18:19033. 10.7448/IAS.18.1.19033 25598476 PMC4297925

[B15] Giunta B. Ehrhart J. Obregon D. F. Lam L. Le L. Jin J. (2011). Antiretroviral medications disrupt microglial phagocytosis of beta-amyloid and increase its production by neurons: implications for HIV-associated neurocognitive disorders. *Mol. Brain* 4:23. 10.1186/1756-6606-4-23 21649911 PMC3128056

[B16] Gougeon M. L. Penicaud L. Fromenty B. Leclercq P. Viard J. P. Capeau J. (2004). Adipocytes targets and actors in the pathogenesis of HIV-associated lipodystrophy and metabolic alterations. *Antivir. Ther.* 9 161–177. 15134178

[B17] Gupta S. Knight A. G. Losso B. Y. Ingram D. K. Keller J. N. Bruce-Keller A. J. (2012). Brain injury caused by HIV protease inhibitors: role of lipodystrophy and insulin resistance. *Antiviral Res.* 95 19–29. 10.1016/j.antiviral.2012.04.010 22580130 PMC3400265

[B18] Harvey J. (2007). Leptin regulation of neuronal excitability and cognitive function. *Curr. Opin. Pharmacol.* 7 643–647. 10.1016/j.coph.2007.10.006 18024215 PMC2635528

[B19] Heaton R. K. Clifford D. B. Franklin D. R. Jr. Woods S. P. Ake C. Vaida F. (2010). HIV-associated neurocognitive disorders persist in the era of potent antiretroviral therapy: CHARTER Study. *Neurology* 75 2087–2096. 10.1212/WNL.0b013e318200d727 21135382 PMC2995535

[B20] Hou X. Xu S. Maitland-Toolan K. A. Sato K. Jiang B. Ido Y. (2008). SIRT1 regulates hepatocyte lipid metabolism through activating AMP-activated protein kinase. *J. Biol. Chem.* 283 20015–20026. 10.1074/jbc.M802187200 18482975 PMC2459285

[B21] Jevtovic D. Vanovac V. Veselinovic M. Salemovic D. Ranin J. Stefanova E. (2009). The incidence of and risk factors for HIV-associated cognitive-motor complex among patients on HAART. *Biomed. Pharmacother.* 63 561–565. 10.1016/j.biopha.2008.09.015 19026516

[B22] Jin J. Grimmig B. Izzo J. Brown L. A. M. Hudson C. Smith A. J. (2016). HIV non-nucleoside reverse transcriptase inhibitor efavirenz reduces neural stem cell proliferation in vitro and in vivo. *Cell Transplant.* 25 1967–1977. 10.3727/096368916X691457 28836850 PMC5683847

[B23] Jones R. Sawleshwarkar S. Michailidis C. Jackson A. Mandalia S. Stebbing J. (2005). Impact of antiretroviral choice on hypercholesterolaemia events: the role of the nucleoside reverse transcriptase inhibitor backbone. *HIV Med.* 6 396–402. 10.1111/j.1468-1293.2005.00325.x 16268821

[B24] Kim J. B. Sarraf P. Wright M. Yao K. M. Mueller E. Solanes G. (1998). Nutritional and insulin regulation of fatty acid synthetase and leptin gene expression through ADD1/SREBP1. *J. Clin. Invest.* 101 1–9. 10.1172/JCI1411 9421459 PMC508533

[B25] Lin Y. J. Chang J. S. Liu X. Tsang H. Chien W. K. Chen J. H. (2015). Genetic variants in PLCB4/PLCB1 as susceptibility loci for coronary artery aneurysm formation in Kawasaki disease in Han Chinese in Taiwan. *Sci. Rep.* 5:14762. 10.1038/srep14762 26434682 PMC4593004

[B26] Ma Q. Vaida F. Wong J. Sanders C. A. Kao Y. T. Croteau D. (2016). Long-term efavirenz use is associated with worse neurocognitive functioning in HIV-infected patients. *J. Neurovirol.* 22 170–178. 10.1007/s13365-015-0382-7 26407716 PMC4783211

[B27] Mason M. M. He Y. Chen H. Quon M. J. Reitman M. (1998). Regulation of leptin promoter function by Sp1. C/EBP, and a novel factor. *Endocrinology* 139 1013–1022. 10.1210/endo.139.3.5792 9492033

[B28] Matoga M. M. Hosseinipour M. C. Aga E. Ribaudo H. J. Kumarasamy N. Bartlett J. (2017). Hyperlipidaemia in HIV-infected patients on lopinavir/ritonavir monotherapy in resource-limited settings. *Antivir. Ther.* 22 205–213. 10.3851/IMP3101 27740537 PMC5392175

[B29] McGregor G. Harvey J. (2017a). Food for thought: leptin regulation of hippocampal function and its role in Alzheimer’s disease. *Neuropharmacology* 136(Pt B), 298–306. 28987937 10.1016/j.neuropharm.2017.09.038

[B30] McGregor G. Harvey J. (2017b). Leptin regulation of synaptic function at hippocampal TA-CA1 and SC-CA1 synapses: implications for health and disease. *Neurochem. Res.* [Epub ahead of print]. 10.1007/s11064-017-2362-1 28819795 PMC6420429

[B31] Miller K. D. Jones E. Yanovski J. A. Shankar R. Feuerstein I. Falloon J. (1998). Visceral abdominal-fat accumulation associated with use of indinavir. *Lancet* 351 871–875. 10.1016/S0140-6736(97)11518-5 9525365

[B32] Moyle G. J. Orkin C. Fisher M. Dhar J. Anderson J. Wilkins E. (2015). A randomized comparative trial of continued abacavir/lamivudine plus efavirenz or replacement with efavirenz/emtricitabine/tenofovir DF in hypercholesterolemic HIV-1 infected individuals. *PLoS One* 10:e0116297. 10.1371/journal.pone.0116297 25658097 PMC4319732

[B33] Mulligan K. Khatami H. Schwarz J. M. Sakkas G. K. Depaoli A. M. Tai V. W. (2009). The effects of recombinant human leptin on visceral fat, dyslipidemia, and insulin resistance in patients with human immunodeficiency virus-associated lipoatrophy and hypoleptinemia. *J. Clin. Endocrinol. Metab.* 94 1137–1144. 10.1210/jc.2008-1588 19174500 PMC2682465

[B34] Nagy G. S. Tsiodras S. Martin L. D. Avihingsanon A. Gavrila A. Hsu W. C. (2003). Human immunodeficiency virus type 1-related lipoatrophy and lipohypertrophy are associated with serum concentrations of leptin. *Clin. Infect. Dis.* 36 795–802. 10.1086/367859 12627366

[B35] Paula A. A. Falcao M. C. Pacheco A. G. (2013). Metabolic syndrome in HIV-infected individuals: underlying mechanisms and epidemiological aspects. *AIDS Res. Ther.* 10:32. 10.1186/1742-6405-10-32 24330597 PMC3874610

[B36] Pedrol E. Llibre J. M. Tasias M. Curran A. Guardiola J. M. Deig E. (2015). Outcome of neuropsychiatric symptoms related to an antiretroviral drug following its substitution by nevirapine: the RELAX study. *HIV Med.* 16 628–634. 10.1111/hiv.12298 26238151

[B37] Pistell P. J. Gupta S. Knight A. G. Domingue M. Uranga R. M. Ingram D. K. (2010). Metabolic and neurologic consequences of chronic lopinavir/ritonavir administration to C57BL/6 mice. *Antiviral Res.* 88 334–342. 10.1016/j.antiviral.2010.10.006 20970459 PMC2991583

[B38] Prot M. Heripret L. Cardot-Leccia N. Perrin C. Aouadi M. Lavrut T. (2006). Long-term treatment with lopinavir-ritonavir induces a reduction in peripheral adipose depots in mice. *Antimicrob. Agents Chemother.* 50 3998–4004. 10.1128/AAC.00625-06 17000748 PMC1693995

[B39] Reagan-Shaw S. Nihal M. Ahmad N. (2008). Dose translation from animal to human studies revisited. *FASEB J.* 22 659–661. 10.1096/fj.07-9574LSF 17942826

[B40] Romao P. R. Lemos J. C. Moreira J. De Chaves G. Moretti M. Castro A. A. (2011). Anti-HIV drugs nevirapine and efavirenz affect anxiety-related behavior and cognitive performance in mice. *Neurotox. Res.* 19 73–80. 10.1007/s12640-009-9141-y 20012242

[B41] Rosen E. D. Spiegelman B. M. (2000). Molecular regulation of adipogenesis. *Annu. Rev. Cell Dev. Biol.* 16 145–171. 10.1146/annurev.cellbio.16.1.145 11031233

[B42] Stauch K. L. Emanuel K. Lamberty B. G. Morsey B. Fox H. S. (2017). Central nervous system-penetrating antiretrovirals impair energetic reserve in striatal nerve terminals. *J. Neurovirol.* 23 795–807. 10.1007/s13365-017-0573-5 28895059 PMC5726941

[B43] Stocker D. N. Meier P. J. Stoller R. Fattinger K. E. (1998). “Buffalo hump” in HIV-1 infection. *Lancet* 352 320–321. 10.1016/S0140-6736(05)60295-4 9690434

[B44] Streck E. L. Ferreira G. K. Scaini G. Rezin G. T. Goncalves C. L. Jeremias I. C. (2011). Non-nucleoside reverse transcriptase inhibitors efavirenz and nevirapine inhibit cytochrome C oxidase in mouse brain regions. *Neurochem. Res.* 36 962–966. 10.1007/s11064-011-0432-3 21365448

[B45] Tiliscan C. Arama V. Mihailescu R. Munteanu D. I. Streinu-Cercel A. Ion D. A. (2015). Leptin expression in HIV-infected patients during antiretroviral therapy. *Germs* 5 92–98. 10.11599/germs.2015.1076 26405677 PMC4570839

[B46] Tsai F. J. Cheng C. F. Lai C. H. Wu Y. C. Ho M. W. Wang J. H. (2017). Effect of antiretroviral therapy use and adherence on the risk of hyperlipidemia among HIV-infected patients, in the highly active antiretroviral therapy era. *Oncotarget* 8 106369–106381. 10.18632/oncotarget.22465 29290955 PMC5739740

[B47] Tsoukas M. A. Farr O. M. Mantzoros C. S. (2015). Leptin in congenital and HIV-associated lipodystrophy. *Metabolism* 64 47–59. 10.1016/j.metabol.2014.07.017 25267014

[B48] Wangpatharawanit P. Sungkanuparph S. (2016). Switching lopinavir/ritonavir to atazanavir/ritonavir vs adding atorvastatin in HIV-Infected patients receiving second-line antiretroviral therapy with hypercholesterolemia: a randomized controlled trial. *Clin. Infect. Dis.* 63 818–820. 10.1093/cid/ciw395 27402817

[B49] Zang M. Xu S. Maitland-Toolan K. A. Zuccollo A. Hou X. Jiang B. (2006). Polyphenols stimulate AMP-activated protein kinase, lower lipids, and inhibit accelerated atherosclerosis in diabetic LDL receptor-deficient mice. *Diabetes Metab. Res. Rev.* 55 2180–2191. 10.2337/db05-1188 16873680

[B50] Zang M. Zuccollo A. Hou X. Nagata D. Walsh K. Herscovitz H. (2004). AMP-activated protein kinase is required for the lipid-lowering effect of metformin in insulin-resistant human HepG2 cells. *J. Biol. Chem.* 279 47898–47905. 10.1074/jbc.M408149200 15371448

[B51] Zhang B. Macnaul K. Szalkowski D. Li Z. Berger J. Moller D. E. (1999). Inhibition of adipocyte differentiation by HIV protease inhibitors. *J. Clin. Endocrinol. Metab.* 84 4274–4277. 10.1210/jcem.84.11.6234 10566684

