# Supplementary figures and images for: Evaluation of Oral Antiretroviral Drugs in Mice With Metabolic and Neurologic Complications
Source: Front Pharmacol. 2018 Sep 4;9:1004. doi: 10.3389/fphar.2018.01004 (PMC6131569; doi:10.3389/fphar.2018.01004)

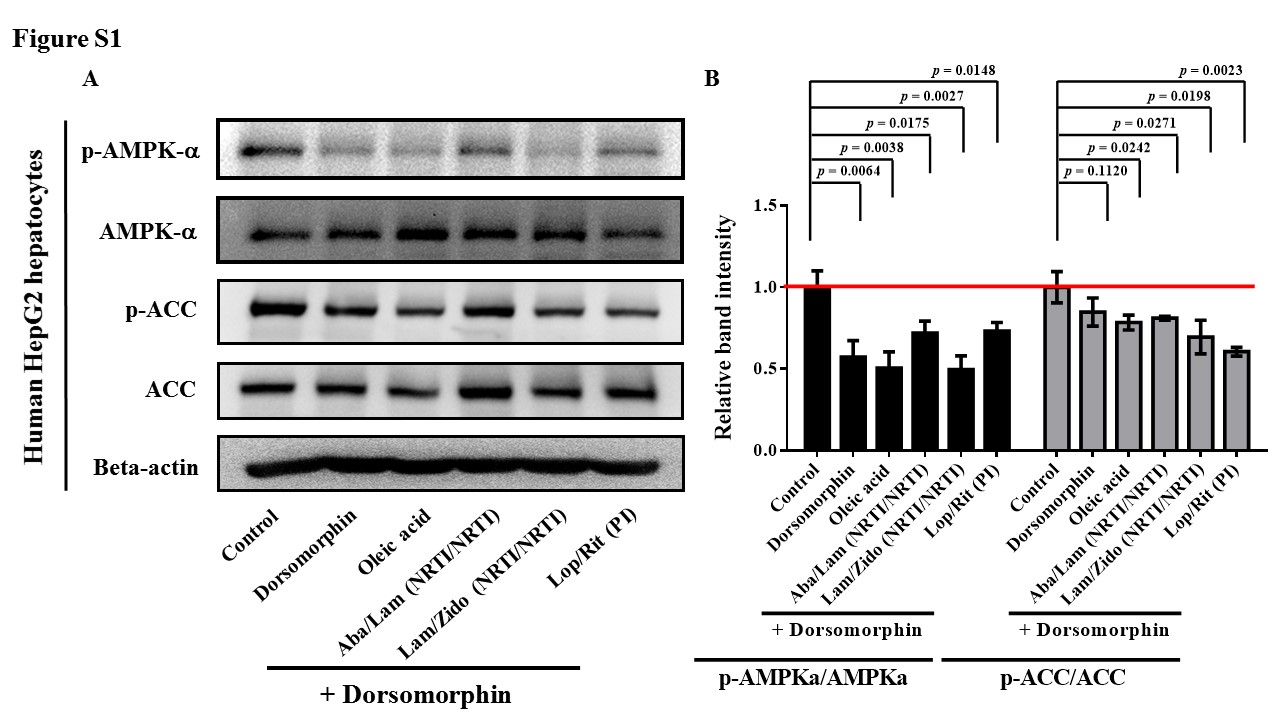

Supplement: FIGURE S1 — Effects of antiretroviral drugs (Aba/Lam, Lam/Zido, and Lop/Rit) with dorsomorphin on lipid metabolism-related AMPK and ACC proteins in human HepG2 hepatocytes. (A) Effects of antiretroviral drugs (Aba/Lam, Lam/Zido, and Lop/Rit) with dorsomorphin on phosphorylation and expression of both AMPKa and ACC proteins by using Western blot analysis. (B) Relative ratios of band intensity of p-AMPKa/AMPKa and p-ACC/ACC when compared with the controls. All data are mean ± SEM for each group. Data are analyzed by the un-paired student t-test in the experimental groups Aba/Lam, Lam/Zido, or Lop/Rit-treated HepG2 cells as compared with the control cells. Dorsomorphin-treated HepG2 cells are used as the AMPK antagonist controls. Oleic acid-treated HepG2 cells are used as the controls to inhibit the AMPK and ACC signaling pathway. [file Image_1.JPEG]
