# Supplementary material for: Evaluation of Oral Antiretroviral Drugs in Mice With Metabolic and Neurologic Complications
Source: Front Pharmacol. 2018 Sep 4;9:1004. doi: 10.3389/fphar.2018.01004 (PMC6131569; doi:10.3389/fphar.2018.01004)
Supplement: TABLE S1 — Demographic characteristics of HIV-1-infected patients with and without neurological disorders. [file Table_1.DOCX]

| **TABLE S1 \| Demographic characteristics of HIV-1-infected patients with and without neurological disorders** | | | |
| --- | --- | --- | --- |
| **Characteristics** | **Neurological disorders** | **Non- neurological disorders** | ***p*-value** |
|  | **N = 3,014** | **N = 12,056** |  |
|  | **N (%)** | **N (%)** |  |
| **Age** |  |  | 0.997 |
| <30 | 1087 ( 36.07%) | 4108 (34.07%) |  |
| 30-40 | 1082 (35.90%) | 4442 ( 36.84%) |  |
| >40 | 845 (28.04%) | 3506 ( 29.08%) |  |
| **Gender** |  |  | 1.000 |
| Male | 2508 ( 83.21%) | 10032 ( 83.21%) |  |
| Female | 506 ( 16.79%) | 2024 ( 16.79%) |  |
| **Comorbidities** |  |  |  |
| Cardio-cerebrovascular diseases | 581 ( 19.28%) | 1705 ( 14.14%) | ***<0.001*** |
| Respiratory diseases | 220 ( 7.3%) | 676 ( 5.61%) | ***<0.001*** |
| Rheumatological diseases | 27 ( 0.9%) | 62 ( 0.51%) | ***0.014*** |
| Digestive diseases | 316 ( 10.48%) | 961 ( 7.97%) | ***<0.001*** |
| Diabetes | 93 ( 3.09%) | 283 ( 2.35%) | ***0.020*** |
| Renal diseases | 50 ( 1.66%) | 77 ( 0.64%) | ***<0.001*** |
| Liver diseases | 234 ( 7.76%) | 761 ( 6.31%) | ***0.004*** |
| Cancer | 36 ( 1.19%) | 112 ( 0.93%) | 0.186 |
|  |  |  |  |
| N, number. | | | |
| *p* values were obtained by the chi-square test. | | | |
| Significant *p-*values (*p* < 0.05) are highlighted in bold italic font. | | | |
| Comorbidities present in the patients prior to their subsequent HIV diagnosis were defined as follows: cardio-cerebrovascular disease (ICD-9-CM: 410, 412, 428, 441, 443.9, 430-438, 785.4, V43.4, and 38.48 (P)), respiratory diseases (ICD-9-CM: 490-496, 500 -505, and 506.4), rheumatic diseases (ICD-9-CM: 710.0, 710.1, 710.4, 714.0-714.2, 714.81, and 725), digestive diseases (ICD-9-CM: 531-534), diabetes (ICD-9-CM: 250.0-250.3, and 250.7), renal disease (ICD-9-CM: 582, 583-583.7, 585, 586, and 588), liver diseases (ICD-9-CM: 571.2, 571.4-571.6, 070.4, 070.5, and 070.7), and cancer (ICD-9-CM: 140-172, 174-195.8, and 200-208). | | | |
