# Supplementary material for: Evaluation of Oral Antiretroviral Drugs in Mice With Metabolic and Neurologic Complications
Source: Front Pharmacol. 2018 Sep 4;9:1004. doi: 10.3389/fphar.2018.01004 (PMC6131569; doi:10.3389/fphar.2018.01004)
Supplement: TABLE S2 — Neurological disorder risk in HIV-1-infected patients according to single type of ART regimen. [file Table_2.DOCX]

| **TABLE S2 \| Neurological disorder risk in HIV-1-infected patients according to single type of ART regimen** | | | | | | | | | | | |
| --- | --- | --- | --- | --- | --- | --- | --- | --- | --- | --- | --- |
| **ART regimen** |  | **Neurological disorder N=3,014** | **Non- neurological disorder N=12,056** |  | **Univariate** | | |  | **Multiple** | | |
|  |  | **N (%)** | **N (%)** |  | **OR** | **95% CI** | ***p* - value** |  | **OR** | **95% CI** | ***p* - value** |
|  |  |  |  |  |  |  |  |  |  |  |  |
| **Non-ART use** |  | 1721 | 7762 |  | 0.71 | (0.66 - 0.78) | ***<.0001*** |  | 0.69 | (0.64 - 0.75) | ***<.0001*** |
| **Single type of ART regimen** |  |  |  |  |  |  |  |  |  |  |  |
| NNRTI |  | 897 | 2790 |  | 1.41 | (1.29 - 1.54) | ***<.0001*** |  | 1.43 | (1.31 - 1.57) | ***<.0001*** |
| NRTI |  | 547 | 1616 |  | 1.43 | (1.29 - 1.59) | ***<.0001*** |  | 1.48 | (1.33 - 1.65) | ***<.0001*** |
| NRTI/NRTI |  | 1180 | 3812 |  | 1.39 | (1.28 - 1.51) | ***<.0001*** |  | 1.42 | (1.30 - 1.55) | ***<.0001*** |
| PI |  | 881 | 2738 |  | 1.41 | (1.29 - 1.54) | ***<.0001*** |  | 1.44 | (1.32 - 1.58) | ***<.0001*** |
| Other ART |  | 14 | 32 |  | 1.88 | (0.99 - 3.54) | 0.0526 |  | 1.87 | (0.99 - 3.55) | 0.0541 |
|  |  |  |  |  |  |  |  |  |  |  |  |
| N, number; ART, antiretroviral therapy; OR, odds ratio; CI, confidence interval; NNRTI, non-nucleoside reverse transcriptase inhibitors; NRTI, nucleoside/nucleotide reverse transcriptase inhibitors; PI, protease inhibitors. | | | | | | | | | | | |
| Adjusted for age, gender, Charlson's comorbidity. | | | | | | | | | | | |
| Significant *p*-values (*p* < 0.05) are highlighted in bold italic font. | | | | | | | | | | | |
| NNRTI includes efavirenz, etravirine, and nevirapine; NRTI includes didanosine, stavudine, abacavir, lamivudine, zidovudine, zalcitabine, tenofovir disoproxil, and emtricitabine; PI includes lopinavir and ritonavir, atazanavir, ritonavir, nelfinavir, indinavir, saquinavir, darunavir, and tipranavir; Other ART includes raltegravir and enfuvirtide. | | | | | | | | | | | |
